# Fetal genetic factors in pregnancy loss: Insights from a meta-analysis and effectiveness of whole exome sequencing

**DOI:** 10.1371/journal.pone.0319052

**Published:** 2025-02-25

**Authors:** Andrea Hadjipanteli, Athina Theodosiou, Ioannis Papaevripidou, Angelos Alexandrou, Nicole Salameh, Paola Evangelidou, Marios Tomazou, Andreas Mavrides, Sozos Fasouliotis, George Anastasiou, Andreas Stavroulis, Niki Agathokleous, Maria Agathokleous, Stelios Tsangarides, Ioannis Kallikas, Kyriakos Kakoullis, Sofia Frakala, Christina Oxinou, Andreas Marnerides, Emilia Athanasiou, Sofia Ourani, Violetta C. Anastasiadou, George Tanteles, Ludmila Kousoulidou, Carolina Sismani

**Affiliations:** 1 The Cyprus Institute of Neurology and Genetics, Cytogenetics and Genomics, Nicosia, Cyprus; 2 ISIS Clinic, Nicosia, Cyprus; 3 Mother and Child Medical Centre, Nicosia, Cyprus; 4 American Medical Center, Nicosia, Cyprus; 5 Limassol Clinic, Limassol, Cyprus; 6 The Fetal Medicine Center Ltd., Limassol, Cyprus; 7 Ledra Clinic, Nicosia, Cyprus; 8 AAK Ultrasound and Fetal Medicine Centre, Nicosia, Cyprus; 9 Apollonion Private Hospital, Nicosia, Cyprus; 10 Christina Oxinou Histopathology/Cytology Laboratory, Nicosia, Cyprus; 11 Guy’s and St Thomas’ NHS Foundation Trust, London, UK; 12 Archbishop Makarios III Hospital, Nicosia, Cyprus; 13 Karaiskakio Foundation, Nicosia, Cyprus; 14 Department of Basic and Clinical Sciences, University of Nicosia Medical School, Nicosia, Cyprus; Human Genetics and Genome Research Institute, National Research Centre, EGYPT

## Abstract

Spontaneous pregnancy loss commonly occurs during the first trimester and can be caused by various factors including chromosomal abnormalities and submicroscopic aberrations. After the first trimester, the etiology of most pregnancy losses remains undetermined. This study aims to fill this gap by an in-depth investigation of the fetal genome and its effect on pregnancy outcome.

Data from 1016 spontaneously aborted fetuses previously referred for genetic testing (2017–2023) were used for meta-analysis. Fetuses were categorized based on gestational age and genetic test result. Additionally, 35 second-third trimester fetuses, that were spontaneously aborted, terminated or died neonatally, with abnormal ultrasounds and unrevealing routine genetic testing were collected. Trio-based whole-exome sequencing was performed for identification of fetal variants that may have caused the pregnancy loss.

The meta-analysis revealed that 822 of 1016 fetuses (80.91%) were aborted during the first trimester, with 569 of 822 (69.22%) successfully diagnosed using conventional genetic testing. The remaining 194 fetuses (19.09%) were aborted during the second-third trimester. Of the 194 second-third trimester aborted fetuses, 163 (84.02%) lacked genetic diagnosis using conventional testing (karyotype and array-CGH). Aneuploidies were the leading cause of spontaneous pregnancy loss in both first and second-third trimester fetuses followed by polyploidies. Thus, the meta-analysis demonstrated that undiagnosed second-third trimester pregnancy losses are more likely to benefit from further genetic investigation.

Application of whole exome sequencing on second-third trimester pregnancy losses, revealed causative variants in 6 of 33 families (18.18%), in genes linked to Mendelian disorders associated with the phenotypes of interest. Pathogenic findings were identified in two additional families in heterozygosity in genes following autosomal recessive inheritance.

Accurate identification of variants in such genes creates new genotype-in utero phenotype associations, with the prospect of new additions in preconception/prenatal diagnostic panels. This study highlights the importance of whole exome sequencing in resolving undiagnosed pregnancy losses.

## Introduction

Pregnancy loss is a common complication, affecting 15% of recognized pregnancies within the first trimester [1,2] while up to 50% of pregnancies are miscarried prior to their clinical recognition [3]. In more rare cases, it can also occur at later stages with one in every 200 pregnancies ending in spontaneous abortion after the second trimester [3]. Pregnancy loss has previously been linked to various causes including environmental, maternal (endometrial abnormalities, autoimmune disorders) and genetic factors [1].

Fetal genetic abnormalities related to spontaneous abortion can have a parental origin or can arise de novo in the embryonic genome [4,5]. Parental – origin genetic abnormalities can result from parental balanced rearrangements such as translocations and inversions. The presence of these balanced rearrangements in the gametes may lead to transmission of derivative chromosomes to the offspring, causing miscarriage and severe congenital malformations [6,7]. Fetal genetic factors associated with spontaneous abortion, especially within the first trimester, are usually large-scale abnormalities such as aneuploidies and polyploidies and other unbalanced structural rearrangements [8]. However, smaller pathogenic genetic changes such as copy number variants (CNVs) and single nucleotide variants (SNVs) can also contribute mostly to second and third trimester pregnancy losses, when identified in genes essential for fetal development [9]. It has been suggested that particular genes have a crucial role in various cellular functions such as cilia biosynthesis and endoplasmic reticulum (ER) stress response and thus in the normal development of the fetus. As a result, improper protein function caused by pathogenic variants within these genes can result in reduced embryonic viability and fetal abnormalities that can affect the course of the pregnancy [10–12]. Advances in ultrasonographic imaging have permitted the visualization of previously undetectable fetal anomalies, suggestive of genetic disorders. Currently, such findings are identified in 2–5% of pregnancies [13] and include cardiac abnormalities, microcephaly, ventriculomegaly, omphalocele, increased echogenicity etc. [14]. Based on several studies, certain ultrasound findings such as small gestational sac, low heart rate of the fetus and oligohydramnios, may indicate a higher probability of spontaneous abortion and embryonic lethality [15].

Although some prenatal markers of genetic abnormality exist, not all ultrasonographic anomalies are suggestive of a particular genetic disorder, mostly due to insufficient reporting of fetal phenotypes in literature and the lack of fetus-specific variant databases [16]. In addition, the phenotype observed in neonates with a genetic disorder can often differ from the one observed prenatally while in other cases, particular characteristics of disorders such as epilepsy cannot be assessed *in-utero*. It is therefore important to report features specific to each developmental stage, thus enriching prenatal phenotypes and facilitating genotype – *in utero* phenotype associations, ultimately enabling more accurate prenatal diagnosis [17,18]. The reporting of pathogenic genetic variants and associated clinical findings is crucial for the enrichment of preconception and prenatal diagnostic panels, as well as for providing guidance to families and physicians for pregnancy management and prognosis [17].

Genetic investigation typically begins with chromosomal analysis, which was previously routinely applied as the first-tier genetic test for the investigation of spontaneous pregnancy loss (SPL) and fetal abnormalities, detecting structural and numerical abnormalities of > 5Mb such as balanced rearrangements and aneuploidies. Array-Comparative Genomic Hybridization (CGH) was introduced to detect copy number changes as small as 30kb [19,20]. Combined, chromosomal analysis and array-CGH can provide a genetic diagnosis in up to 40% of cases, leaving the majority unresolved [21].

More recently developed Next Generation Sequencing (NGS) is gradually being incorporated in the genetic investigation of prenatal diagnosis and SPL, offering the ability to detect SNVs and indels [22]. Thus far, NGS was mostly being utilized in the postnatal setting, with studies showing that whole-exome sequencing (WES) used postnatally for unresolved cases has a diagnostic yield of 20–80% [17,23]. Challenges in the interpretation of variants especially variants of uncertain significance (VUS) have delayed the incorporation of NGS in the prenatal diagnostic setting. However, studies have demonstrated that prenatal WES diagnostic yield ranges from 6.2% to 57.1% depending on the inclusion criteria. Diagnostic yield is influenced by the affected organ system, the number of affected organ systems, the extend of phenotypic description as well as the availability of parental samples [18]. Usually, a lower diagnostic yield is observed in studies including fetuses with non-specific ultrasound abnormalities, in contrast to those that only include fetuses with extensive and highly specific phenotypes [17].

Ultimately, WES can be a valuable tool for prenatal diagnosis in cases that remained unresolved after chromosomal analysis and array-CGH [24]. Accurate diagnosis can assist the couple and referring physician in making an informed decision regarding the pregnancy and prenatal treatment/newborn care. The chance of recurrency in future pregnancies can also be estimated, saving the parents from emotional turmoil [24]. The participation of multidisciplinary teams is of high importance for creating new genotype-*in utero* phenotype associations to improve our understanding of the prenatal manifestation of genetic disorders. With the incorporation of WES in prenatal diagnostic pipelines, better prognosis will be possible, leading to a considerable decrease in the rate of unexplained fetal death [13,21,25].

This study presents a meta-analysis including 1016 fetuses previously referred for conventional genetic analysis. The results obtained from the meta-analysis were compared with previously published research, in an aim to identify the group of fetuses that would benefit the most from additional methods of genetic testing such as WES. This study also aimed to obtain data from 33 families undergoing WES to define its role in prenatal diagnosis and to uncover previously unidentified variants associated with embryonic/newborn lethality and genes essential for normal fetal development.

## Materials and methods

### Patients and samples

This study, including the corresponding consent forms, was approved by the Cyprus National Bioethics Committee (ΕΕΒΚ/ΕΠ/2020/38) and written informed consent was obtained from all participating families, allowing the use of their biological samples and publication of results.

### Study design

This study was designed as a two-part investigation. A retrospective meta-analysis was carried out, examining data from 1016 spontaneously aborted fetuses to evaluate the gestational age at the time of loss and identify genetic abnormalities related to pregnancy loss at various developmental stages. Additionally, a cohort study was performed on 33 families with pregnancy loss and/or abnormal prenatal sonographic findings that had unrevealing routine genetic testing, implementing WES to identify potential genetic contributors*.*

### Meta-analysis

Results obtained from genetic testing of 1016 spontaneously aborted fetuses (SAFs) referred to the department of Cytogenetics and Genomics of the Cyprus Institute of Neurology and Genetics due to pregnancy loss were extracted from the departmental database. The samples had all previously undergone chromosomal analysis and/or array-CGH and Quantitative Fluorescent (QF)-PCR as well as other forms of targeted testing. This data was accessed on the 23^rd^ of October 2020. Authors and researchers had no access to information that could identify individual participants. The samples were then categorised based on 1) trimester of pregnancy at the time of pregnancy loss, 2) normal vs. abnormal genetic test result and 3) the type of abnormality identified in each sample with abnormal genetic test result. The data was then analysed to identify the group of fetuses/families that would benefit the most from undergoing futher investigation with WES.

### WES inclusion criteria

Based on the database meta-analysis, prenatal samples including amniotic fluid, chorionic villi or skin biopsies were collected from second-third trimester fetuses with abnormal ultrasound findings and/or prenatal death, referred to our laboratory for genetic investigation. The fetuses had all previously undergone some form of genetic testing, including chromosomal analysis and array-CGH with no causative findings. Recruitment of participating families started on the 2^nd^ of November 2020 and ended on the 26^th^ of September 2023. In total, 33 families provided written informed consents to be enrolled in this cohort to undergo trio-based WES. All clinical features identified in the fetuses were described using Human Phenotype Ontology (HPO) terms [26].

### Genetic investigation

Fetal DNA was isolated from amniotic fluid using QIAGEN QIAamp DNA Mini Kit. Parental DNA was also isolated from peripheral blood using QIAGEN QIAamp DNA Blood Midi Kit according to the manufacturer’s instructions.

Library preparation was performed with Illumina DNA Prep with Enrichment kit and exome sequencing was carried out using Illumina NextSeq 500/550 High Output Kit v2.5 on Illumina NextSeq 500 System as per the manufacturer’s protocol. After completion of the WES run, trio check was carried out using Peddy software package v0.4.2 [27]to ensure genetic relatedness between the fetus and parents. Fastq files were loaded on commercial VarSome Clinical Saphetor SA platform [28] for alignment to hg19 reference genome, variant calling and annotation. SNVs, small variants and CNVs were predicted from the platform. Variants were filtered based on minor allele frequency (MAF), inheritance mode, coverage (>8 at the specific locus) and genomic location (variants targeted in coding and splicing regions ± 10 base pairs). The pathogenicity of each variant was assessed based on the recommendations of ACGS 2020 guidelines (Ellard et al., 2020), ClinGen sequence variant interpretation working group and was assisted by the automatic scoring ACMG classification of VarSome Clinical and VarSome. Variants classified as pathogenic, likely pathogenic and variants of unknown significance (VUS) with strong evidence of pathogenicity, were prioritized as they are more likely to have an effect on the protein. Conservation scores were also taken into account since evolutionary conserved regions of the genome tend to be of high importance for cellular function. Τhe phenotypic features of the probands were compared against other literature reports of either the same variant or different variants within the same gene. Variants were reported in cases where the proband’s phenotype matched the phenotype observed in other patients carrying variants in the same gene. Pathogenic and likely pathogenic variants that fit the fetal phenotype were reported back to the patients and referring physicians. VUS were only reported if there was some evidence of pathogenicity and/or they were identified in trans with a pathogenic or likely pathogenic variant in a gene following autosomal recessive inheritance. CNV analysis was also carried out using CNVkit and custom scripts in R to identify CNVs with a resolution of 100kb [29].

For variant validation, bidirectional Sanger sequencing was employed, using specifically designed primers (Metabion- Munich, Germany) through the Primer3 web interphase tool flanking the mutation site [30–32]. Applied Biosystems™ BigDye™ Terminator v1.1 Cycle Sequencing Kit (ThermoFisher Scientific- Waltham, Massachusetts, USA) and Affymetrix ExoSAP-IT^®^† (Fisher Scientific- Pittsburgh, Pennsylvania, USA) were used for this procedure.

## Results

### Meta-analysis

**Fig 1:**
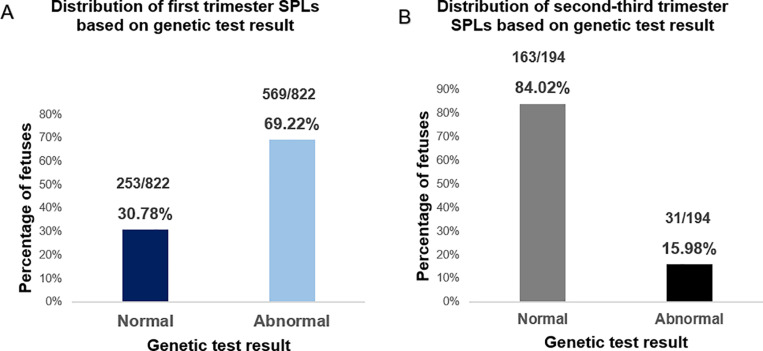
(A) Bar chart showing the distribution of genetic test result in first trimester SAF. In total 253 out of 822 (30.78%) first trimester aborted fetuses had a normal genetic test result and 569 of 822 (69.22%) first trimester aborted fetuses had an abnormal genetic test result (B) **Bar chart showing the distribution of genetic test result in second-third trimester spontaneously aborted fetuses.** In total 163 out of 194 (84.02%) second-third trimester aborted fetuses had a normal genetic test result and 31 of 194 (15.98%) second-third trimester aborted fetuses had an abnormal genetic test result SPL=spontaneous pregnancy loss.

Initially, 1016 SAFs previously referred for routine genetic testing (chromosomal analysis, array-CGH, QF-PCR and targeted testing) were selected from the departmental database. Out of 1016 samples, 822 (80.91%) fetuses were spontaneously aborted during the first trimester of pregnancy and only 194 (19.09%) fetuses were spontaneously aborted during the second-third trimesters of pregnancy. The majority of first trimester SAFs (n = 569, 69.22%) had an abnormal genetic test result which determined the cause of the SPL, while the rest (n = 253, 30.78%) had a normal genetic test result leaving the cause of SPL undetermined (Fig 1). In contrast, a substantially higher percentage of second-third trimester SAFs (84.02%, n = 163) remained genetically undiagnosed. Meanwhile only 15.98% (n = 31) of fetuses from this group received a genetic diagnosis consistent with the reason for pregnancy loss using conventional methods of genetic testing. Thus, the majority of second-third trimester pregnancy losses do not receive a diagnosis relevant to the reason of pregnancy loss using chromosomal analysis and array-CGH.

Out of all first trimester aborted fetuses with an abnormal genetic test result, the most commonly identified genetic abnormality was aneuploidy or mosaic aneuploidy. More specifically, aneuploidies were detected in a total of 455 out of 569 first trimester SAFs with an abnormal genetic test (79.96%) (Table 1). The most common aneuploidy detected in first trimester SAFs was monosomy X with a total of 90 fetuses, followed by trisomies 16 (n = 74) and 22 (n = 74). The rarest aneuploidies observed in this group of SAFs were trisomy Y (n = 1), trisomy 11 (n = 2) and trisomy 17 (n = 2). Aneuploidies involving the chromosomes 1, 5 and 19 were not identified. Aneuploidies were also detected in 20 out of 31 (64.52%) of second-third trimester aborted fetuses with an abnormal genetic result, making it the most frequently identified abnormality in this group of fetuses as well. Trisomy 21(n = 7) was most commonly identified in this group of fetuses, followed by trisomy 13 (n = 4). Monosomy X which was the most common aneuploidy in first trimester aborted fetuses was identified 3 times in second-third trimester SAFs along with trisomy 18. A single case of trisomy 10, trisomy 14, and trisomy Y was also detected. Polyploidies were also detected in both groups of SAFs. In particular, 95 out of 569 first trimester fetuses with an abnormal genetic test (16.7%) were spontaneously aborted as a result of a polyploidy. In total, six of 31 (19.35%) second-third trimester fetuses with an abnormal genetic result were aborted due either of the following polyploidies: 69,XXX or 69,XXY. Unbalanced structural abnormalities in full or mosaic form had also been identified in a total of 12 fetuses. Out of these, 11 were spontaneously aborted during the first trimester of pregnancy accounting for 1.93% of first trimester aborted fetuses with an abnormal genetic result and one was spontaneously aborted in the second-third trimester of pregnancy accounting for 3.23% of second-third trimester aborted fetuses with an abnormal genetic result. CNVs have been determined to be the reason of pregnancy loss in seven of 569 fetuses miscarried during the first trimester (1.23%), and four of 31 fetuses in the second-third trimester (12.9%). Finally, only one case of pregnancy loss caused by uniparental disomy (UPD) has been identified in this study in a fetus spontaneously aborted during the first trimester of pregnancy (0.18%) (Table 1).

**Table 1.** Distribution of abnormalities identified on genetic testing per trimester of pregnancy.

Type of abnormality	Number of 1^st^ trimester SAFs	Number of 2^nd^-3^rd^ trimester SAFs
**Aneuploidy/Mosaic aneuploidy**	79.96% (455 of 569)	64.52% (20 of 31)
**Polyploidy/Mosaic polyploidy**	16.7% (95 of 569)	19.35% (6 of 31)
**Unbalanced structural abnormalities/Mosaic unbalanced structural abnormalities**	1.93% (11 of 569)	3.23% (1 of 31)
**CNVs**	1.23% (7 of 569)	12.9% (4 of 31)
**UPD**	0.18% (1 of 569)	0% (0 of 31)

### Genetic investigation with WES

**Fig 2:**
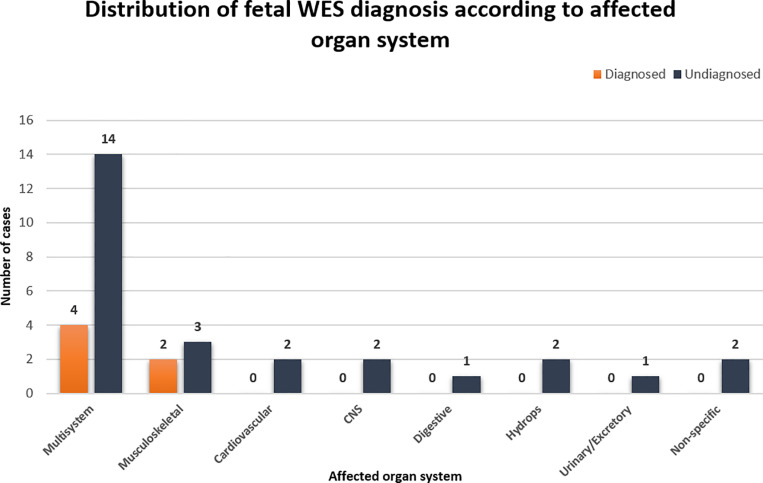
Bar chart showing the distribution of WES diagnosis according to the affected organ system. Orange bars indicate the diagnosed cases and blue bars indicate the undiagnosed cases. CNS=Central nervous system. Non-specific abnormalities include low birth weight and IUD..

In total, 35 probands from 33 families were referred for genetic analysis using chromosomal analysis, array-CGH and WES after IUD/termination of pregnancy. The probands of each family were on average 23.58 weeks of gestation at the time of sample collection and referral for genetic analysis, and all of them were referred either during the second or third trimester of pregnancy. Amniotic fluid, chorionic villi and skin biopsies were collected from fetuses with phenotypic abnormalities in various organ systems including musculoskeletal (n = 5), cardiovascular (n = 2), central nervous (CNS) (n = 2), digestive (n = 1), urinary/excretory (n = 1), hydrops abnormalities (n = 2) and non-specific abnormalities including low birth weight and IUD (n = 3). More than half of the referred fetuses presented multisystem anomalies (n = 18) which is the presence of at least one major abnormality in two or more different organ systems (Fig 2). Out of the 33 families, six (18.18%) had a causative finding that was previously linked to a disorder relevant to the prenatal phenotype, which also followed the expected inheritance pattern. Out of these six diagnosed families, four fetuses were referred with multisystem anomalies and two fetuses were referred with musculoskeletal abnormalities. In addition to the six families that received a diagnosis from WES, pathogenic findings possibly implicated in the fetal phenotype were identified in two more families in heterozygosity in genes following an autosomal recessive inheritance pattern (Table 2). All three fetuses in these two families presented with multisystem anomalies (Fig 2).

**Table 2.** List of prenatal cases for which findings relevant to the reason of referral were identified.

Family No.	Gestational age in weeks at the time of referral	Sex	Phenotype	Affected gene	Variant	Novel/ known	Coding impact	Variant classification	Inheritance/ zygosity	OMIM diagnosis
**1** (33)	22	F	**Prenatally:** hand clenching (HP:0001188), ventriculomegaly (HP:0002119), aplasia of the gallbladder (HP:0011466), pericardial effusion (HP:0001698), intrauterine growth retardation (HP:0001511)	***SCN2A***	NM_001040143.2:c.751G>A, p.(Val251Ile)	**De novo:** Known	Missense	**Pathogenic** (PS2—strong; PS4—moderate; PM1—moderate; PP3—moderate; PM2—supporting)	Autosomal dominant / de novo, heterozygous	Developmental and epileptic encephalopathy 11- AD (OMIM #613721)
			**Postnatal patient follow-up:** epileptic spasms (HP:0011097), skeletal muscle atrophy (HP:0003202) refractory status epilepticus (HP:0032867), polymicrogyria (HP:0002126)/lissencephaly (HP:0001339), neonatal death (HP:0003811)							
**2** (33)	31.4	M	**Prenatally:** Ddecreased fetal movement (HP:0001558), talipes equinovarus of the left foot (HP:0001762), talipes calcaneovarus of the right foot (HP:0001884), polyhydramnios (HP: 0001561), neonatal death (HP:0003811)	***SCN4A***	**Paternally inherited:** NM_000334.4:c.4340T>C, p.(Phe1447Ser),	**Paternally inherited:** Novel	**Paternally inherited:** Missense	**Paternally inherited: Likely pathogenic** (PP3—strong; PM1—moderate; PP3—moderate; PM2—supporting)	Autosomal recessive / compound heterozygous	Severe fetal congenital myopathy 22B- AR (OMIM# 620369)
			**Post mortem examination:** single transverse palmar crease (HP:0000954), anteverted nares (HP:0000463), pulmonary hypoplasia (HP:0002089), narrow thorax (HP:0000774), low-set ears (HP:0000369), high arched palate (HP:0000218), bilateral diaphragmatic eventration (HP:0009110), disproportionality of the head with the anterior-posterior axis of the skull vault being disproportionately greater than the transverse axis and the head appearing large in relation to the trunk and limbs, reduced muscle bulk of the limbs and the psoas muscles with myopathic-type histologic changes on microscopic examination of various skeletal muscles		**Maternally inherited:** NM_000334.4:c.3798G>C p.(Glu1266Asp)	**Maternally inherited:** Known	**Maternally inherited:** Missense	**Maternally inherited:** Likely pathogenic (PP3—moderate; PM2—supporting; PP4—supporting)		
**3**	30.6	F	IUGR (HP:0001511), polyhydramnios (HP:0001561), hypokinesia (HP:0002375), clenched hands (HP:0001188), intrauterine death (HP:0034241)	***KLHL40***	NM_152393.4:c.1305C>A, p.(Tyr435Ter)	Known	Nonsense	**Likely pathogenic** (PVS1—Very strong, PM2—Supporting)	Autosomal recessive / homozygous	Nemaline myopathy 8- AR (OMIM #615348)
**4**	22	F	Polyhydramnios (HP:0001561), hypokinesia (HP:0002375), clenched hands (HP:0001188), talipes (HP:0001883), termination of pregnancy	***RYR1***	**Paternally inherited:** NM_000334.4:c.6721C>T, p.(Arg2241Ter)	**Paternally inherited:** Known	**Paternally inherited:** Nonsense	**Paternally inherited:** Pathogenic (PVS1—Very strong, PM3—Strong, PM2—Supporting)	Autosomal recessive / compound heterozygous	Congenital myopathy 1B- AR (OMIM #255320)
					**Maternally inherited:** NM_000540.3:c.8068-3C>G	**Maternally inherited:** Novel	**Maternally inherited:** Splicing defect	**Maternally inherited:** VUS (PM2—Supporting, PP3—Supporting		
**5**	13.5	M	Bowed long bones /telephone receiver-like deformity (HP:0006487), short long bones (HP:0003026), termination of pregnancy	***FGFR3***	**De novo:** NM_000142.5:c.746C>G, p.(Ser249Cys)	Known	Missense	**Pathogenic** (PS2—Strong, PS4—Strong, PM2—Supporting, PP3—Strong)	Autosomal dominant / de novo, heterozygous	Thanatophoric dysplasia, type I- AD (OMIM #187600)
**6**	22	F	Polydactyly (HP:0010442), inferior cerebellar vermis hypoplasia (HP:0007068)	***CPLANE1***	**Paternally inherited:** NM_001384732.1:c.8633-4_8633-3del	**Paternally inherited:** Known	**Paternally inherited:** Splicing defect	**Paternally inherited:** VUS (PM2—supporting, PP3—supporting)	Autosomal recessive / heterozygous	Joubert syndrome 17- AR (OMIM #614615)
					**Maternally inherited:** NM_001384732.1:c.1819del p.(Tyr607ThrfsTer6)	**Maternally inherited:** Known	**Maternally inherited:** Frameshift	**Maternally inherited:**Likely pathogenic (PVS1—very strong, PM2—supporting)		
**7**	29	M	Ventriculomegaly (HP:0002119), abnormal cerebellum morphology (HP:0001317), pleural effusion (HP:0002202), ascites (HP:0001541), hydrocele testis (HP:0000034), polyhydramnios (HP:0001561), neonatal death (HP:0003811)	***USP18***	**Maternally inherited**: NM_017414.4:c.772C>T, p.(Arg258Ter)	Novel	Nonsense	**Likely pathogenic** (PVS1—Very strong, PM2—Supporting)	Autosomal recessive / heterozygous	Pseudo-TORCH syndrome 2- AR (OMIM #617397)
**8**	13	F	Hexadactyly (HP:0100259), encephalocele (HP:0002084), polycystic kidney dysplasia (HP:0000113)	***CC2D2A***	**Paternally inherited:** NM_001080522.2:c.3055C>T p.(Arg1019Ter)	Known	Nonsense	**Pathogenic** (PVS1—Very strong, PM2—Supporting)	Autosomal recessive / heterozygous	Meckel syndrome 6- AR (OMIM #612284)
		M								

In this cohort, nine causative variants relevant to the phenotype of interest were identified in families 1–6. In addition to these findings, two possibly causative heterozygous variants relevant to the phenotype of interest were identified in families 7 and 8. All findings were identified in genes known to cause Mendelian disorders. The variants were classified as pathogenic or likely pathogenic based on ACGS 2020 guidelines, with the exception of the maternally inherited variant of family 4 and the paternally inherited variant of family 6 that were classified as VUS. Overall, four missense, four nonsense, two splicing and one frameshift variant were reported in families 1–8. Out of all identified variants, eight had previously been reported in ClinVar or relevant literature and three were novel. All identified variants that were reported as causative had a strong correlation with the referred fetus’ phenotype based on OMIM and relevant literature. The detailed clinical picture identified in the fetus of each family along with information relevant to the candidate variants can be found in Table 2. The remaining 25 families that were enrolled in this study did not receive a genetic diagnosis even after WES. All families that had no causative findings identified using karyotype, array-CGH and WES are presented in S1 Table.

The findings of families 1 and 2 have been described in detail in a previous publication [33].

#### Family 1.

Trio-based WES analysis revealed the presence of a de novo, pathogenic, missense variant in exon 8 of the *SCN2A* (OMIM #182390) gene in the proband. The variant was reported twice in ClinVar as pathogenic in a patient with Developmental and Epileptic Encephalopathy 11 (DEE11) (RCV001329203) (SCV001520577.1) and a patient with Epileptic Encephalopathy (RCV000416970) (SCV000494509.1).

#### Family 2.

The proband of family 2 died as soon as the umbilical cord was cut at delivery, which took place at 36 weeks.

Two variants were identified in gene *SCN4A* (OMIM #603967), in a compound heterozygous state in the proband using trio-based WES analysis. The paternally inherited missense variant (SCV004015158.1) is absent from gnomAD population databases and considered to be novel as it has not been previously reported in any frequency databases. A nearby maternally inherited missense variant (SCV004015159.1) is also absent from gnomAD population databases, however, has been reported in homozygosity a cohort study in a patient with myopathic changes [34].

In a subsequent pregnancy of the couple, DNA was isolated from amniotic fluid, which was collected at 16.6 weeks of gestation. The second fetus was female and was referred for genetic investigation with Sanger sequencing which confirmed the presence of the previously identified findings in the proband, in the absence of any phenotypic abnormalities. The pregnancy was later terminated at 24 weeks of gestation due to the identification of severe structural abnormalities at 22 weeks. Pediatric post-mortem examination of the second fetus revealed structural abnormalities almost identical with the proband.

#### Family 3.

WES analysis revealed a likely pathogenic nonsense variant (rs573886282, SCV004015157.1) in exon 2 of gene *KLHL40* (OMIM #615340) located in 3p22.1, in the spontaneously aborted fetus. The variant was present in a heterozygous state in both parents and a homozygous state in the proband. This is a null variant in a gene where loss-of-function is a known mechanism of disease and was absent from local screening databases. Pathogenicity was also supported by eight Meta *In-silico* prediction scores including BayesDel noAF. The assigned CADD score was 40. The variant was not previously reported in ClinVar and no relevant publications were found, however was reported in gnomAD twice.

#### Family 4.

WES analysis revealed the presence of a compound heterozygous variant. A paternally inherited pathogenic heterozygous nonsense variant (SCV005091224) was identified in *RYR1* gene (OMIM #180901), located in 19q13.2 in the proband. The variant is located in exon 41 of the gene *RYR1* (rs200563280). This is a null variant in a gene where loss-of-function is a known mechanism of disease. In addition, eight Meta in-silico prediction scores also support the pathogenicity of the variant including BayesDel noAF. The variant has previously been submitted in ClinVar as pathogenic 16 times, as likely pathogenic once and as VUS once. Relevant publications were also found [35]. A nearby novel maternally inherited splicing variant (SCV005091225) was also identified and classified as VUS. The variant has not been identified in any frequency databases and was also absent from GnomAD population databases. Although eight meta in-silico prediction tools support a deleterious effect of the variant, the evidence available is not sufficient to confidently support its pathogenicity.

#### Family 5.

CVS from the fetus was referred for WES analysis during the 13th week of gestation due to a suspicion of a skeletal dysplasia. WES analysis revealed a de novo heterozygous missense variant (SCV005091226, rs121913483) in exon 7 of gene *FGFR3* (OMIM * 134934) located in 4p16.3 which was classified as pathogenic. The missense variant was found in a heterozygous state in the proband while both parents were homozygous for the wild type allele. This variant has previously been reported multiple times in ClinVar as pathogenic and has been associated with thanatophoric dysplasia type 1 in several publications [36,37]. Pathogenicity was also supported by 11 Meta in-silico prediction scores including BayesDel noAF, REVEL and MetaSVM. The assigned CADD and PhyloP100way scores were 25.2 and 7.427 respectively.

#### Family 6.

WES analysis revealed two variants in compound heterozygous state in the proband in the gene CPLANE1 (OMIM * 614571), located in 5p13.2. A maternally inherited heterozygous one base deletion (SCV005091227, rs777686211) was identified in exon 12 of *CPLANE1*, leading to a premature termination 6 bases downstream the deletion. The change was classified as likely pathogenic. This is a null variant in a gene where loss-of-function is a known mechanism of disease and was found in extremely low frequency in GnomAD (ƒ =  0.000179 in gnomAD Exomes). The variant had previously been reported in ClinVar by 15 submitters as pathogenic/likely pathogenic. A second paternally inherited splicing two base deletion (SCV005091228, rs34646696) within *CPLANE1* was identified in the proband. The change was classified as VUS. It was found in extremely low frequency in GnomAD (*f* =  0.0000393 in gnomAD Exomes) while computational tools support a deleterious effect. The variant had not been previously reported in ClinVar. However, a single base deletion and duplication have been reported in the same region in The Single Nucleotide Polymorphism database (dbSNP) suggesting variability at the specific locus. These alterations have been classified as benign/likely benign.

#### Families 7 and 8.

Pathogenic/likely pathogenic variants were identified in the probands of families 7 and 8 in heterozygosity, in genes following an autosomal recessive inheritance. WES revealed the presence of a novel nonsense likely pathogenic variant (SCV005091229) in the gene *USP18* in the fetus of family 7. Family 8 was referred with a monochorionic diamniotic twin pregnancy. WES revealed the presence of the same known nonsense pathogenic variant (SCV005091230) in both fetuses in the gene *CC2D2A*.

*USP18* and *CC2D2A* are genes that follow an autosomal recessive inheritance pattern. Therefore, although the prenatal clinical presentation identified in the fetuses, was in concordance with the existing prenatal phenotype of each corresponding gene, a single finding is not sufficient to cause an abnormal phenotype. Coverage reports of exonic regions were generated for the gene of interest in each of these two families, to assess the possibility of a second variant located within an insufficiently covered region of the gene. Based on the coverage reports, all regions in the genes *USP18* and *CC2D2A* appeared to be sufficiently covered thus excluding this possibility.

## Discussion

### Meta-analysis

The process of patient selection and the inclusion criteria established for each study, have a significant effect on subsequent WES detection rate [17,18]. It is therefore important to target the groups of fetuses that would benefit the most from WES and would have a higher chance of receiving a diagnosis. A meta-analysis of the departmental database was carried out, aiming to provide data that would aid in the selection of this target group, by investigating previous pregnancy loss referrals and their diagnostic outcomes.

Overall, the result of our meta-analysis was in agreement with previous research which had shown that approximately 50%–70% of SAFs present some form of cytogenetic abnormality with the most common being autosomal trisomies and monosomy X (approximately 80% of the cases combined) and polyploidies (20%) [3]. In our study, 59.06% of all the fetuses had an abnormal genetic test result of which aneuploidies appeared to be the leading cause of pregnancy loss (79.96% in the first and 64.52% in second-third trimester).

Chromosomal abnormalities tend to vary depending on the gestational age at the time of fetal demise. For example, miscarriages observed during early developmental stages often involve uncommon aneuploidies, whereas pregnancy losses occurring at more advanced developmental stages tend to present chromosomal abnormalities more commonly seen in live births [38]. Consistent with the above studies, we observed that the most common aneuploidy in first trimester fetuses was monosomy X (19.78% of all identified aneuploidies), followed by trisomies 16 and 22. As expected, fetuses presenting trisomies 16 and 22 were spontaneously aborted during the first trimester as these aneuploidies are incompatible with life. In contrast, the aneuploidies identified in the second-third trimester aborted fetuses were primarily trisomies 13, 18 and 21. Only a singular case of trisomies 10 and 14 were identified in second-third trimester aborted fetuses confirming the statement that unusual aneuploidies are not largely detected in more advanced developmental stages.

Following aneuploidies, polyploidies were the second most common abnormality identified in the referred fetuses. Both digynic and diandric polyploidies were identified in first trimester pregnancy losses, including 69,XXX, 69,XXY, 69,XYY, 92,XXYY, and 92,XXXX. In contrast, only 69,XXX and 69,XXY had been identified in second-third trimester losses. Although the origin of the second X chromosome in cases with 69,XXY was not assessed, the most probable scenario is that it is of maternal origin, as fetuses with diandric polyploidies are usually aborted during early stages of pregnancy [39].

Despite the high detection rate of combined chromosomal analysis and array-CGH for first-trimester pregnancy losses, 84.02% of spontaneously aborted second-third trimester fetuses do not receive a genetic diagnosis. A portion of these undiagnosed cases could be attributed to environmental factors such as maternal endometrial abnormalities and chronic conditions. In terms of genetic etiology, studies have shown that pregnancy losses occurring at more advanced developmental stages are more likely to be caused by smaller genetic abnormalities such as CNVs, indels and SNVs that cannot be detected by conventional methods of genetic testing and require additional genetic investigation [40,41]. Unlike fetuses with large chromosomal abnormalities that are incompatible with life and spontaneously aborted during the first trimester of pregnancy, fetuses with smaller genetic aberrations may be aborted at later developmental stages or even be born with phenotypic abnormalities that vary in severity, depending on the nature of the genetic abnormality and the effect it has on protein level [41]. For instance, a higher percentage of second-third trimester pregnancy losses (12.9%) in this study were caused by an CNV compared to first trimester pregnancy losses (1.23%).

The above data leads to the conclusion that, in combination with the ability of ultrasound to provide a more detailed and comprehensive phenotypic description in second-third trimester fetuses compared to those in the first trimester, the former group would derive the greatest benefit from WES in the absence of a previous genetic diagnosis. Therefore, a small study was conducted in which undiagnosed second-third trimester fetuses underwent WES to evaluate its utility for such cases.

Our WES investigation, along with prior research, highlights how the inclusion criteria can greatly impact the diagnostic yield. Previous studies show a 26% diagnostic yield when selecting cases of prenatal or perinatal deaths with thorough phenotypic data from autopsies. In contrast, studies focused solely on fetal structural abnormalities identified via ultrasound yield significantly lower (8.5%–10%) [42]. Here, the diagnostic yield, which was calculated at 18.18% (excluding fetuses from families 7, and 8), surpassed the 8.5%–10% anticipated when focusing solely on structural abnormalities. However, due to the absence of detailed phenotypic reporting in some cases and the lack of autopsies in most cases, the diagnostic yield fell short of the expected 26%. Therefore, in addition to patient inclusion criteria, successful WES diagnosis is also heavily dependent on detailed and accurate prenatal phenotypic descriptions [18].

### WES findings

In this study, several inheritance patterns and types of variants were identified using WES. Out of eight families with findings related to the phenotype of interest, six families had findings in genes following an autosomal recessive inheritance and two had findings in genes with an autosomal dominant inheritance that occurred de novo. This displays the necessity of family-based WES, especially for countries like Cyprus that have a small population and thus an increased chance of recessive disorders.

In the aforementioned eight families, 11 causative variants were identified, out of which eight had previously been reported in publicly available databases and three were novel. The reporting of novel variants in this study was mainly based on previous phenotypic descriptions relevant to the gene in which the variant of interest was identified. The successful diagnosis of these families was therefore dependent on previous causative variant reporting and the associated clinical picture described in the literature, reflecting the importance of enriching the prenatal phenotype of disorders as well as creating new genotype-phenotype associations. The absence of suitable databases and guidelines for fetal variant classification creates difficulties and disparities in diagnostic yield between different studies [42]. Although a more detailed phenotypic description can be obtained at later stages of pregnancy, a major challenge of prenatal diagnosis is the fact that the fetal phenotype of many disorders has not yet been described or may considerably vary from the corresponding postnatal phenotype. Reporting phenotypic abnormalities of disorders with prenatal onset is therefore of utmost importance to create valuable additions to preexisting phenotypic spectrums, confirm previous findings and thus assist health care professionals in recognizing various disorders prenatally [43].

Variants that have been characterized in the literature before and have previously been established as a cause of a genetic disorder were much more easily identified and reported than novel variants. As a result, the diagnosis was made in a shorter time span, allowing families more time for genetic counselling and family planning. For example, the variant identified in the fetus of family 1 [33] in the *SCN2A* gene, had previously been reported as pathogenic and had been associated with developmental and epileptic encephalopathy 11 (DEE11) (OMIM #613721) which expedited the diagnosis. DEE11 is a neurologic condition characterized by seizures that appear neonatally [44]. In the case of family 1, postnatal phenotypic characteristics suggestive of DEE11 were identified by the referring physician. However, the lack of reported prenatal manifestations in the literature would create difficulties with reaching a diagnosis antenatally had the variant not previously been reported. Nevertheless, in this instance, new correlations between genotype and prenatal phenotype were established, potentially aiding healthcare providers in managing similar prenatal cases in the future.

In some cases, interpreting genetic variants was challenging due to the discrepancies between prenatal and postnatal phenotypic manifestations of genetic disorders. In addition, several cases in this cohort did not present extensive phenotypic abnormalities either due to gestational age or imaging limitations, creating even more complications in the interpretation of variants and therefore diagnosis. For instance, causative variants in *RYR1* were detected in the fetus of family 4, leading to the diagnosis of congenital myopathy 1B (OMIM #255320). The fetus presented polyhydramnios, clenched hands, hypokinesia and talipes on ultrasound. Although these phenotypic features are suggestive of a congenital myopathy, they are not specific to one myopathy, which can create uncertainties in cases where the candidate variants are classified as VUS or in cases where the phenotypic abnormalities could be a result of several candidate variants. In this case the pregnancy had been terminated due to the abnormalities identified on ultrasound. However, it is plausible that more disorder-specific phenotypic traits could have emerged at more advanced developmental stages. In such cases, fetopsy could also provide valuable insights into disorder-specific manifestations that can often not be assessed due to imaging limitations. Monitoring fetal phenotypic changes throughout pregnancy, neonatally, and post-mortem is crucial, as disorder-specific abnormalities usually manifest as the pregnancy progresses. In this cohort fetopsy was only employed in the fetuses of family 2. Prenatal characteristics identified on ultrasound in this case, were sufficient for the successful diagnosis of congenital myopathy 22B (OMIM #620369) which was assisted by a cohort in which prenatal phenotypic manifestations were reported [45]. Nevertheless, the autopsies conducted on the fetuses in family 2 not only validated the diagnosis but also uncovered valuable additions to the existing phenotypic spectrum of this myopathy, ultimately aiding healthcare providers in its diagnosis [33]. In these cases, where the phenotypic spectrum of a disorder has been expanded, WES failed to reveal any additional candidate variants that would account for the atypical features of the suspected genetic condition. However, the possibility of dual diagnosis cannot be ruled out since additional candidate variants may have been overlooked due to filtering or human error.

The most evident genotype-phenotype associations were observed in families 3 and 5. In family 3, the fetus displayed Intrauterine growth retardation (IUGR), polyhydramnios, hypokinesia, and clenched hands, all of which are features suggestive of myopathy. A candidate variant, classified as likely pathogenic, was found in homozygosity in the gene *KLHL40* in the fetus, leading to the diagnosis of nemaline myopathy 8 (OMIM #615348), which is inherited in an autosomal recessive manner. In line with previous discoveries in fetuses with pathogenic variants in *KLHL40*, which commonly lead to either prenatal or neonatal demise, the fetus in family 3 died prenatally [46,47]. This variant had previously been reported in patients diagnosed with nemaline myopathy 8, providing additional evidence for the diagnosis. In family 5, the fetus exhibited characteristic features of thanatophoric dysplasia type I (TDI) (OMIM #186700), including shortened and bowed long bones, also known as a “telephone-receiver like deformity.” The candidate variant (Ser249Cys) which was identified in the gene *FGFR3,* is one of five known disease-causing variants associated with thanatophoric dysplasia type I, thereby leading to a diagnosis [36,48].

It is also important to note that initially, the paternally inherited variant in the *CPLANE1* gene of family 6 was not reported due to insufficient evidence supporting its pathogenicity. However, upon re-analysis, the variant was reclassified as a VUS with more significant evidence of pathogenicity. This highlights the value of re-analyzing WES data in cases with partial or no findings, as variants of interest may be reclassified as new information emerges.

Potentially causative heterozygous variants have been identified in the genes *USP18* and *CC2D2A* in the fetuses of families 7 and 8 respectively. Although the variants appear to be associated with the corresponding phenotype of each fetus, these genes typically follow an autosomal recessive mode of inheritance, thus a single causative hit would not be sufficient to cause an abnormal phenotype. The presence of a second pathogenic hit within these genes, which has not been identified by array-CGH and WES analysis cannot be excluded. This could be due to unsuccessful variant calling by the analysis platform, a cryptic indel or structural variant or a variant not classified as pathogenic. Unsuccessful variant calling due to inadequate coverage has been excluded, since all the regions within this gene have been sufficiently covered and manually inspected. These cases could be subjected to additional methods of investigation such as targeted gene long-read sequencing and whole genome sequencing techniques for the identification of previously unidentified causative variants.

Importantly, similarly with the results of previous studies, it was observed that the fetuses receiving a WES diagnosis in this cohort exhibited either multisystem or musculoskeletal anomalies [16,49,50]. This study therefore provides additional confirmation that WES analysis of prenatal cases without causative findings from chromosomal analysis and array-CGH is more likely to uncover the genetic cause of pregnancy loss when the fetal phenotype consists of multisystem or skeletal anomalies [50]. Fetuses presenting phenotypic abnormalities isolated to other organ systems could potentially benefit more from targeted forms of genetic testing such as NGS panels.

### Challenges and pitfalls of prenatal WES

Overall, studies have shown that WES is a highly valuable tool for postnatal diagnosis and is slowly being incorporated into the prenatal diagnostic setting as well, despite the existing obstacles [24]. A main challenge of prenatal WES is turnaround time (TAT). Although this was not a factor to be taken into consideration in this study since all pregnancies ended in IUD/termination or neonatal death prior to their enrolment, timely WES is essential in prenatal diagnosis to allow time for decision making [49]. Incidental findings relevant to the fetus and parents also complicate the use of WES in prenatal diagnosis. Participants in this study could opt in or out of being informed of such findings. One mother was found to have a known frameshift pathogenic *BRCA1* variant (NM_007300.4:c.3018_3021del) relating to breast and ovarian cancer familial 1 (AD), emphasizing the need for strict regulations and consent forms to manage incidental findings and avoid delays in diagnosis.

Prior research in Cyprus shows WES has a 43% diagnostic yield for postnatal patients with multiple malformation syndrome (MMS) [51]. However, in this study, only 22.22% (4/18) of fetuses with multisystem anomalies received a diagnosis, likely due to incomplete phenotypic reporting and imaging limitations. Fetopsies could improve diagnostic yield by revealing traits undetectable via ultrasound. Accurate diagnosis requires detailed prenatal phenotypic reporting, highlighting the need for databases cataloging fetal-specific variants and phenotypes, as prenatal manifestations often differ from postnatal ones.

## Conclusions

This study suggests the use of WES for the genetic diagnosis of second-third trimester SAFs with an unrevealing karyotype and array-CGH result. In agreement with previous research, the diagnostic power of prenatal WES in fetuses with skeletal and multisystem anomalies is highlighted in this study [49,52], while novel variants in fetuses with abnormal sonographic findings have also been reported. As a result, new genotype-phenotype associations have been created which can ultimately facilitate prenatal diagnosis, and genetic counselling, allowing for possible treatments and early interventions. This study has also contributed to the enrichment of the prenatal phenotypic spectrum of various disorders and emphasized the vital role of a detailed prenatal clinical description in patients undergoing WES [43], as well as the use of post-mortem examinations where possible.

## Supporting information

S1 Table List presenting all the cases (families) undergoing WES. (DOCX)

## Abbreviations

ACGS: Association for clinical genetic science
ACMG: American college of medical genetics and genomics
AD: Autosomal dominant
AR: Autosomal recessive
CGH: Comparative genomic hybridization
CNV: Copy number variant
CNS: Central nervous system;
DEE11: Developmental and epileptic encephalopathy 11
ER: Endoplasmic reticulum
FGFR3: Fibroblast growth factor receptor 3
HPO: Human phenotype ontology
IUD: Intrauterine death
MMS: Multiple malformation syndrome
NGS: Next-generation sequencing
OMIM: Online mendelian inheritance in man
QF-PCR: Quantitative fluorescent polymerase chain reaction
SAF: Spontaneously aborted fetus/fetuses
SNV: Single nucleotide variant
SPL: Spontaneous pregnancy loss
TDI: Thanatophoric dysplasia type I.
TAT: Turnaround time
UPD: Uniparental disomy
VUS: Variant of uncertain significance
WES: Whole exome sequencing;
IUGR: , Intrauterine growth retardation.
