# Supplementary material for: Fetal genetic factors in pregnancy loss: Insights from a meta-analysis and effectiveness of whole exome sequencing
Source: PLoS One. 2025 Feb 25;20(2):e0319052. doi: 10.1371/journal.pone.0319052 (PMC11856309; doi:10.1371/journal.pone.0319052)
Supplement: S1 Table — (DOCX) [file pone.0319052.s001.docx]

| **Family No.** | **Gestational age (weeks)** | **Sex** | **Reason for referral** | **Previous testing** |
| --- | --- | --- | --- | --- |
| **F1** | 22 | F | **Prenatally**: hand clenching (HP:0001188), ventriculomegaly (HP:0002119), aplasia of the gallbladder (HP:0011466), pericardial effusion (HP:0001698), intrauterine growth retardation (HP:0001511)  **Postnatal patient follow-up:** epileptic spasms (HP:0011097), skeletal muscle atrophy (HP:0003202) refractory status epilepticus (HP:0032867), polymicrogyria (HP:0002126)/lissencephaly (HP:0001339) | Normal karyotype, array-CGH:  Paternally inherited gain in a region of unknown significance unrelated to the reason of referral ((Xp11.21(55442023_55932729)x3 pat) |
| **F2** | 31.4 | M | **Prenatally**: Decreased foetal movement (HP:0001558), talipes equinovarus of the left foot (HP:0001762), talipes calcaneovarus of the right foot (HP:0001884), polyhydramnios (HP: 0001561)  **Post mortem examination:** single transverse palmar crease (HP:0000954), anteverted nares (HP:0000463), pulmonary hypoplasia (HP:0002089), narrow thorax (HP:0000774), low-set ears (HP:0000369), high arched palate (HP:0000218), bilateral diaphragmatic eventration (HP:0009110), disproportionality of the head with the anterior-posterior axis of the skull vault being disproportionately greater than the transverse axis and the head appearing large in relation to the trunk and limbs, reduced muscle bulk of the limbs and the psoas muscles with myopathic-type histologic changes on microscopic examination of  various skeletal muscles | Normal karyotype, array-CGH: maternally inherited deletion in Prader-Willi region  ((15q11.2(22765628_23300287)x1 mat) |
| **F3** | 30.6 | F | IUGR (HP:0001511), polyhydramnios (HP:0001561), hypokinesia (HP:0002375), clenched hands (HP:0001188), intrauterine death (HP:0034241) | Normal karyotype, normal array-CGH |
| **F4** | 22 | F | Polyhydramnios (HP:0001561), hypokinesia (HP:0002375), clenched hands (HP:0001188), talipes (HP:0001883), termination of pregnancy | Normal karyotype, normal array-CGH |
| **F5** | 13.5 | M | Bowed long bones /telephone receiver-like deformity (HP:0006487), short long bones (HP:0003026), termination of pregnancy | Normal karyotype, normal array-CGH |
| **F6** | 22 | F | Polydactyly (HP:0010442), inferior cerebellar vermis hypoplasia (HP:0007068) | Normal karyotype, normal array-CGH |
| **F7** | 29 | M | Ventriculomegaly (HP:0002119), abnormal cerebellum morphology (HP:0001317), pleural effusion (HP:0002202), ascites (HP:0001541), hydrocele testis (HP:0000034), polyhydramnios (HP:0001561), neonatal death (HP:0003811) | Normal karyotype, normal array-CGH |
| **F8** | 13 | F | Hexadactyly (HP:0100259), encephalocele (HP:0002084), polycystic kidney dysplasia (HP:0000113) | Normal karyotype, normal array-CGH |
|  |  | M |  |  |
| **F9** | 26 | M | Hydrops fetalis (HP:0001789), foetal pleural effusion (HP:0025676), termination of pregnancy | Normal karyotype, normal array-CGH |
| **F10** | 39 | M | IUD (HP:0034241) | Normal karyotype, normal array-CGH |
| **F11** | 37.4 | M | IUD (HP:0034241), hydrops fetalis (HP:0001789) | Paternally inherited 1Mb interstitial deletion (4p15.1-4p14 (35071123-36044115)) |
| **F12** | 16 | M | IUD (HP:0034241), hydrops fetalis (HP:0001789) | Normal karyotype, normal array-CGH |
|  | 29 | M | IUD (HP:0034241), severe hydrocephalus (HP:0006882) | Normal karyotype, normal array-CGH |
| **F13** | 21 | F | Congenital cardiac abnormalities, echogenic fetal bowel (HP:0010943), overlapping toes (HP:0001845), disproportion of the three-vessel view, hypoplastic aortic arch (HP:0012304), aortic isthmus hypoplasia (HP:0034227), suspicion of coarctation of aorta (HP:0001680), ventricular septal defect (HP:0001629), tapering fingers (HP:0001182), brachydactyly (HP:0001156), mild coarse facial features (HP:0000280) | Normal karyotype, normal array-CGH |
| **F14** | 24 | F | IUD (HP:0034241), agenesis of corpus callosum (HP:0001274), pulmonary stenosis (HP:0001642), oligohydramnios (HP:0001562), severe intrauterine growth retardation (HP:0001511), abnormal cerebellar vermis morphology (HP:00002334), microcephaly (HP:0000252), possible double outlet right ventricle (HP:0001719), Blake’s pouch cyst (HP:0033140) | Normal karyotype, normal array-CGH |
| **F15** | 23 | M | Complex congenital cardiac abnormalities, double outlet right ventricle (HP:0001719), severe pulmonary artery hypoplasia (HP:0004971), truncus arteriosus (HP:0001660), persistent left superior vena cava (HP:0005301) | Normal karyotype, normal array-CGH |
| **F16** | 22.3 | F | IUD (HP:0034241), right sided cleft lip and palate(?), increased nuchal translucency (HP:0010880), left hand syndactyly (HP:0006101), skeletal dysplasia (HP:0002652), almost full amputation of both legs (HP:0001218), amniotic constriction ring (HP:0009775) | Normal karyotype, normal array-CGH |
| **F17** | 22 | M | Abnormal urinary tract (HP:0000079), enlarged kidney (HP:0000105), abnormal renal echogenicity (HP:0033130), distended bladder (HP:0025487), termination of pregnancy | Normal karyotype, normal array-CGH |
| **F18** | 22.1 | F | Tetralogy of Fallot (HP:0001636), pulmonary artery hypoplasia (HP:0004971), ventricular septal defect (HP:0001629) | Normal karyotype, normal array-CGH |
| **F19** | 20.6 | F | Skeletal dysplasia (HP:0002652), campomelic dysplasia, bowing of the long bones (HP:0006487), short foetal femur length (HP:0011428), short foetal humerus length (HP:0011429), peroneal absence (HP:0009049?), low set ears (HP:0000369), abnormal earlobe morphology (HP:0000363), microcephaly (HP:0000252), aplasia of the fibula (HP:0006492), termination of pregnancy | Normal karyotype, normal array-CGH |
| **F20** | 38 | M | IUD (HP:0034241) | Normal karyotype |
| **F21** | 18 | F | Increased nuchal translucency (HP:0010880), renal dysplasia (HP:0000110), polycystic kidney dysplasia (HP:0000113), oligohydramnios (HP:0001562) | Normal karyotype, normal array-CGH |
| **F22** | 24 | F | IUD (HP:0034241), anal atresia (HP:0002023), aplasia of the gallbladder (HP:0011466), foetal bowel dilation (HP:4000140) | Normal karyotype, normal array-CGH |
| **F23** | N/A | F | Neonatal death (HP:0003811), respiratory insufficiency (HP:0002093), pulmonary hypertension (HP:0002092), pneumothorax (HP:0002107), upslanting palpebral fissures (HP:0000582), wide nasal bridge (HP:0000431), full lips (HP:0012471), short columella (HP:0002000), short chin (HP:0000331) | Normal array-CGH |
| **F24** | 35 | F | IUD (HP:0034241), increased nuchal translucency (HP:0010880), small for gestational age (HP:0001518) | Normal karyotype, normal array-CGH |
| **F25** | 24 | M | Selective intrauterine growth retardation (HP:0001511), single umbilical artery (HP:0001195), hypoplasia of the cerebellar vermis (HP:0006817), Blake’s pouch cyst (HP:0033140) (Monochorionic Diamniotic twins) | Normal karyotype, normal array-CGH |
|  |  | M | Apparently phenotypically normal | Normal karyotype, normal array-CGH |
| **F26** | 21 | F | Microcephaly (HP:0000252), agenesis of corpus callosum (HP:0001274), small cisterna magna, hypoplasia of the cerebellum (HP:0007360), hydronephrosis (HP:0000126), cleft lip (HP:0000202), cleft palate (HP:0000175), brachycephaly (HP:0000248) | Normal karyotype, normal array-CGH |
| **F27** | N/A | M | Congenital anomalies of the kidney and urinary tract (as a baby), bladder exstrophy (HP:0002836), epispadias (HP:0000039), micropenis (HP:0000054), short stature (HP:0004322), depressed nasal bridge (HP:0005280), low set anteriorly rotated ears (HP:0000369)( HP:0000411), long philtrum (HP:0000343), up slanted palpebral fissures (HP:0000582), mild retrognathia (HP:0000278), bulbous nasal tip (HP:0005274), anteverted nares HP:0000463), deep palmar crease (HP:0006191), anteriorly placed anus (HP:0001545) | Normal karyotype, normal array-CGH |
| **F28** |  | F | Omphalocele (HP:0001539), lower limb dysmetria (HP:0020035), macroglossia (HP:0000158), hemivertebrae (HP:0002937), aortopulmonary collateral arteries (HP:0031834) | Normal array-CGH |
| **F29** | 18.2 | M | Prenatal death (HP:0034241), intrauterine growth retardation (HP:0001511), ventricular septal defect (HP:0001629), left aortic arch with aberrant right subclavian artery (HP:0031055), abnormality of the middle posterior fossa (HP:0000932), vermian hypoplasia (HP:0001320), low set posteriorly rotated ears (HP:0000368), fine lips (HP:0000159), large mouth (HP:0000154), triangular nose (HP:0000451), anteverted nares (HP:0000463), down slanted palpebral fissures (HP:0000494) | Normal karyotype, normal array-CGH |
| **F30** | 13.3 | F | Aplasia of the ulna (HP:0003982), absent radius (HP:0003974), hypoplastic femur (HP:0005613) | Normal karyotype, normal array-CGH |
| **F31** | 21.5 | F | Agenesis of corpus callosum (HP:0001274), termination of pregnancy | Normal karyotype, familial 20p11.22p11.21 duplication identified on array-CGH |
| **F32** | 21.6 | M | Placental abruption (HP:0011419), talipes equinovarus (HP:0001762), hypoplasia of the cerebellar vermis (HP:0006817), Dandy-Walker malformation (HP:0001305), single umbilical artery (HP:0001195), anhydramnios (HP:0025700), intrauterine growth retardation (HP:0001511), hypotonia (HP:0001252), overlapping toe (HP:0001845), ventricular septal defect (HP:0001629), respiratory failure (HP:0002878) , failure to thrive (HP:0001508),  single transverse palmar crease (HP:0000954), wide anterior fontanel (HP:0000260), wide nasal bridge (HP:0000431), prominent nasal tip (HP:0005274), low-set, posteriorly rotated ears (HP:0000368) | Normal karyotype, de novo 1p31.1(77579537_78454754) duplication identified on array-CGH. The region was not relevant to the phenotype |
| **F33** | 21 | M | Polydactyly (HP:0010442), distal shortening of limbs (HP:0006402), termination of pregnancy | Normal karyotype |
